# Exercise training in pulmonary arterial hypertension associated with connective tissue diseases

**DOI:** 10.1186/ar3883

**Published:** 2012-06-18

**Authors:** Ekkehard Grünig, Felicitas Maier, Nicola Ehlken, Christine Fischer, Mona Lichtblau, Norbert Blank, Christoph Fiehn, Frank Stöckl, Felix Prange, Gerd Staehler, Frank Reichenberger, Henning Tiede, Michael Halank, Hans-Jürgen Seyfarth, Simone Wagner, Christian Nagel

**Affiliations:** 1 Centre for Pulmonary Hypertension at Thoraxclinic Heidelberg, Thoraxclinic at the University Hospital Heidelberg, Amalienstraße 5, Heidelberg, 69126, Germany; 2 Department of Human Genetics, University of Heidelberg, Im Neuenheimer Feld 366, Heidelberg, 69120, Germany; 3 Department of Rheumatology, University of Heidelberg, Im Neuenheimer Feld 410, Heidelberg, 69120, Gemany; 4 Department of Rheumatology, ACURA Centre for Rheumatic Diseases, Rotenbachtalstraße 5, Baden-Baden, 76530, Germany; 5 Department of Internal Medicine, Medical Clinic III, Grafenstraße 9, Darmstadt, 64283, Germany; 6 Centre for Pulmonary Hypertension, Clinic Löwenstein, Geißhölzle 62, Löwenstein, 74245, Germany; 7 Department of Pneumology, University Hospital Gießen, Klinikstraße 33, Giessen, 35392, Germany; 8 Department of Pneumology, University Hospital Dresden, Fetscherstraße 74, Dresden, 01307, Germany; 9 Department of Pneumology, University Hospital Leipzig, Liebigstraße 20, Leipzig, 04103, Germany; 10 Department of Neurology, University Hospital Heidelberg, Im Neuenheimer Feld 400, Heidelberg, 69126, Germany

## Abstract

**Introduction:**

The objective of this prospective study was to assess short- and long-term efficacy of exercise training (ET) as add-on to medical therapy in patients with connective tissue disease-associated pulmonary arterial hypertension (CTD-APAH).

**Methods:**

Patients with invasively confirmed CTD-APAH received ET in-hospital for 3 weeks and continued at home for 12 weeks. Efficacy parameters have been evaluated at baseline and after 15 weeks by blinded-observers. Survival rate has been evaluated in a follow-up period of 2.9 ± 1.9 years.

**Results:**

Twenty-one consecutive patients were included and assessed at baseline, and after 3 weeks, 14 after 15 weeks. Patients significantly improved the mean distance walked in 6 minutes compared to baseline by 67 ± 52 meters after 3 weeks (p < 0.001) and by 71 ± 35 meters after 15 weeks (p = 0.003), scores of quality of life (p < 0.05), heart rate at rest, peak oxygen consumption, oxygen saturation and maximal workload. Systolic pulmonary artery pressure and diastolic systemic blood pressure improved significantly after 3 weeks of ET. The 1- and 2-year overall-survival rates were 100%, the 3-year survival 73%. In one patient lung transplantation was performed 6 months after ET.

**Conclusion:**

ET as add-on to medical therapy is highly effective in patients with CTD-APAH to improve work capacity, quality of life and further prognostic relevant parameters and possibly improves the 1-, 2- and 3-year survival rate. Further randomized controlled studies are needed to confirm these results.

**Trial registration:**

ClinicalTrials.gov: NCT00491309.

## Introduction

Pulmonary arterial hypertension (PAH) is characterized by increased pulmonary arterial pressure and pulmonary vascular resistance [1] and can be associated with connective tissue disease (CTD) such as systemic sclerosis (SSc) [2] systemic lupus erythematosus (SLE) [3,4] and mixed connective tissue diseases (MCTD) [5]. Associated PAH (APAH) accounts for approximately half of all patients with PAH [6] and has identical histological findings as idiopathic PAH (IPAH). However, patients with CTD-APAH seem to have a more severely affected clinical phenotype than patients with IPAH. In the American Registry to Evaluate Early and long Term PAH Disease Management (REVEAL Registry), patients with CTD-APAH had a significantly lower 6-minute walking distance (6MWD), higher B-type natriuretic peptide levels, lower diffusing capacity of carbon monoxide and a lower 1-year survival rate than patients with IPAH [7]. Despite advances in PAH treatment, CTD-APAH continues to be progressive, with a 1-year on-treatment mortality of approximately 12.5 to 17.0% compared to 5.0 to 10.0% in IPAH [7,8]. In comparison to other forms of APAH, patients with SSc-APAH demonstrated the worst 1-, 2- and 3-year survival rates of 78.0%, 58.0% and 47.0%, respectively [9]. Patients with CTD-APAH showed a significantly reduced time to hospitalization, had a higher mean age at diagnosis and higher incidence of comorbidities such as renal insufficiency and Raynaud's phenomenon [7,10]. Furthermore, randomized controlled studies reveal reduced efficacy of PAH-targeted medication in this subgroup. For example, in the BREATHE-1 study, bosentan therapy improved baseline 6MWD by 46 meters in 102 patients with IPAH, but only by 3 meters in the 33 patients with SSc-APAH [11]. In addition, therapy with ambrisentan resulted in significant improvement in the 6MWD among patients with IPAH but not with CTD-APAH [12]. Thus, patients with CTD-APAH in particular, may have a high need for additional therapeutic tools to address their exercise capacity, quality of life (QoL) and survival.

Exercise training (ET) has shown beneficial effects on exercise capacity and QoL in patients with PAH [13-15], SLE [16] and in SSc [17-19]. ET also improved peak oxygen consumption and World Health Organization functional class (WHO-FC) in patients with pulmonary hypertension [13], and possibly clinical outcomes, with 1- and 2-year survival rates of 100 and 95%, respectively [14]. Up to now there has been no study focusing on the effect of exercise training in patients with CTD-APAH. The aim of this study was to prospectively assess the effects of ET on prognostic relevant factors such as 6MWD and QoL, and to analyze the survival rate in a cohort of patients with CTD-APAH.

## Materials and methods

### Study population and design

This prospective study investigated patients with CTD-APAH who received exercise and respiratory training as an add-on to disease-targeted medication, between October 2007 and July 2011. Further inclusion criteria were that patients must be aged between 18 and 80 years and classified as WHO-FC II to IV. Patients had to be under optimized medical therapy for PAH (endothelin-antagonists, inhaled or parenteral prostanoids, phosphodiesterase-5-inhibitors, anticoagulants, diuretics, and supplemental oxygen) and for the underlying rheumatologic disease (prednisone, methotrexate) for at least 2 months before entering the study. The diagnosis of CTD-APAH was established at the participating pulmonary hypertension (PH) centers according to current guidelines [1,15]. The rheumatologic diagnosis of each patient had been confirmed by specialized rheumatologic centers. Patients with severe interstitial lung disease were excluded from the study. All patients underwent a detailed clinical work up including right heart catheterization, and all gave written informed consent for this study, which was approved by the Ethics Committee of the University of Heidelberg.

### Outcome measures

Efficacy parameters of ET were prospectively assessed at baseline, week 3, and week 15 as described previously [13,14]. The 6MWD was carried out under standardized conditions [20]. Cardiopulmonary exercise testing and stress Doppler echocardiography were performed during supine bicycle exercise as described previously [13]. Systolic pulmonary artery pressure (sPAP), systolic (RRsys) and diastolic (RRdiast) systemic blood pressure, workload, heart rate, ventilation, oxygen uptake (VO_2_), oxygen pulse (VO_2_/heart rate), oxygen saturation and carbon dioxide output (VCO_2_) were evaluated continuously. The anaerobic threshold (AT) was detected with the V-slope method. Gas exchange, Borg dyspnea index (with 6 representing no exertion and 20 maximal exertion) [21] and changes in WHO-FC were analyzed after 3 and 15 weeks. Health-related QoL assessment was measured by the Short Form Health Survey questionnaire (SF-36) [22]; the baseline scores were compared to the results after 15 weeks. The 6MWD and analysis of the SF-36 questionnaire were performed by investigators who were blinded to the patients' clinical data, so that the investigators did not know the values at any time point.

### Exercise training program

We performed a program especially developed for patients with PH, that comprised at least 1.5 h/day ET as described previously [13,14]. The program was started for the first 3 weeks in the Rehabilitation Clinic, Koenigstuhl, Heidelberg. It consisted of interval bicycle ergometer training at low workloads, mental gait training, dumbbell-training of single muscle groups using low weights (500 to 1000 g) and respiratory therapy 5 days/week. The training was continued at home with at least 30 minutes/day at 5 days/week for the following 12 weeks. Patients were asked to continue with ET at home after the 15-weeks-visit. In addition to physical training patients received psychological support and performed mental training to improve their perception of individual physical abilities and limits. Physicians specialized in rehabilitation medicine and PH experts closely supervised the training program as described before [13,14]. Adverse events were recorded whenever they occurred. Oxygen saturation and heart rate were monitored continuously throughout the training and used to adjust the training intensity. When patients' oxygen saturation fell below 90% during exercise they received supplemental oxygen (3 to 10 L/min) throughout the training. At discharge from hospital after 3 weeks, patients received an individualized training manual and ordered a bicycle ergometer for use at home. All patients were asked to keep close contact with the physicians involved in the training program, and with their general practitioners and the specialized center.

### Follow-up assessment

In 2011 all participating patients were interviewed by telephone using a half-structured questionnaire. The patients were asked about their current symptoms for WHO-FC, if and how they pursued ET at home, whether they had any adverse events from ET, any further cardiac events that might have occurred since the last observation and current medication. Patients who did not attend the last 15-week follow-up visit were the reason for missing the examination. If the patient was deceased, date of death was recorded and their relatives and/or treating physicians were asked for the cause and circumstances of death.

### Statistical methods

The analyses were performed by a statistician (CF). The within-group comparisons of baseline and weeks 3 and 15 for 6MWD, workload, Borg dyspnea index, parameters of gas exchange, systolic pulmonary arterial pressure (sPAP), systemic blood pressures, heart rate as well as summation and sub-scores of the SF-36 were compared by the Wilcoxon rank test. Comparison of WHO-FC at different time points was performed by the McNemar-Bowden test. All tests were two sided and *P*-values < 0.05 were considered statistically significant. Bonferroni adjustment for multiple comparisons was performed for the primary endpoints, 6MWD and QoL parameters; *P*-values < 0.005 were considered significant. All analyses were carried out using IBM SPSS V19 (IBM Corp. Armonk, NY, USA). We describe in detail, the patients who did not attend the 15-week measurement. To access the consequences for the main efficacy parameter, 6MWD, we performed multiple imputations of 6MWD at 15 weeks within SPSS using the predictive mean matching model, with age, peak oxygen consumption, oxygen consumption at the AT, oxygen saturation at maximal workload, systolic blood pressure at maximal workload at baseline, 6MWD at baseline and 3 weeks, and pain subscale of the SF-36 at baseline as explanatory variables. Kaplan-Meier estimates were used for survival analysis with the 95% two-sided confidence interval (CI) calculated using Greenwood's formula. All treated patients were included in the survival analysis. Patients who died or underwent organ transplantation were counted as endpoints; survivors were regarded as censored.

## Results

### Study population

We included 22 consecutive patients (Table 1). One patient had to be excluded because she could not perform the examinations after week 3 and week 15, due to upper airway infection. Thus, the final study group consisted of 21 patients: 9 patients (43%) with SSc-APAH, 7 (33%) with SLE-APAH, 2 (10%) with MCTD and 3 (14%) with other CTDs such as Jo-1 syndrome and Sjöegren's syndrome. Demographic data, diagnosis, functional class, hemodynamic values, lung function and medical therapy in the full study population are summarized in Table 1. At baseline 9 patients (43%) were in WHO-FC II, 7 (33%) were classified as WHO-FC III and 5 (24%) as WHO-FC IV. Combination therapies, including two to three PAH-targeted agents, were used in 62% of patients (Table 1).

**Table 1:** Baseline characteristics

Patients, number		21	
Gender male/female	1	/	20
Age, years	52	±	18
Height, cm	165	±	6
Weight, kg	68	±	11
**WHO functional class, number (%)**			
II	9	(43)	
III	7	(33)	
IV	5	(24)	
**Lung function**			
TLCO, %	55	±	18
FEV1%VC, %	78	±	7
pO2, mmHg	69	±	12
**Mean 6-minute-walking distance, meter**			
	386	±	121
**Differential Diagnosis, number (%)**			
Systemic sclerosis	8	(43%)	
Systemic lupus	7	(33%)	
MCTD	2	(10%)	
Others	4	(14%)	
**Cardiac catherization**			
Pulmonary artery pressure, mmHg	49	±	13
Pulmonary vascular resistance, dyn×sec×cm-5	789	±	498
Right atrium pressure, mmHg	7	±	3
Pulmonary artery oxygen saturation, %	62	±	10
PCWP, mmHg	10	±	5
Cardiac index, L×min×m^-2^	2.6	±	0.6
**PAH-targeted medication, number (%)**			
Endothelin receptor antagonists	12	(57%)	
Phosphodiesterase-5-Inhibitors	15	(71%)	
Prostanoids inhaled	3	(14%)	
Prostanoids intravenous	1	(5%)	
Calcium channel blockers	2	(10%)	
Soluble guanyl cyclase-stimulator	3	(14%)	
**Combination therapy, number (%)**			
Monotherapy	8	(38%)	
Dual therapy	10	(48%)	
Triple therapy	3	(14%)	

Results are presented as mean ± standard deviation unless stated otherwise

WHO: World Health Organization; FEV1%VC: forced expiratory volume in one second/vital capacity; TLCO: carbon monoxide transfer factor; pO2: partial oxygen pressure; PCWP: pulmonary capillary wedge pressure; MCTD: mixed connective tissue disease.

### Assessment of training effects

**Figure 1:**
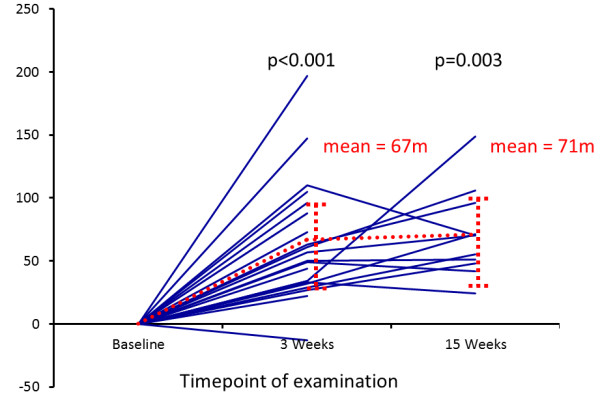
**Individual changes in six-minute-walking distance (6MWD) after 3 and 15 weeks exercise training**. Using the Wilcoxon rank test, *P *< 0.001 was obtained for the comparisons with baseline at week 3 (*n *= 21) and *P *= 0.003 for comparison to week 15 (*n *= 11). The dashed line indicates the mean change from baseline in 6MWD (67 ± 52 meters and 72 ± 35 meters).

ET significantly improved the 6MWD from 386 ± 121 (mean ± standard deviation (SD)) meters by 64 ± 47 meters after 3 weeks (*P *< 0.001) and by 71 ± 35 meters after 15 weeks (*P *< 0.003) (Figure 1). All patients except one improved their 6MWD (Figure 1). In the patient with no increase in 6MWD the test was limited by her hip joint osteoarthritis. However, in this patient other parameters of physical exercise capacity improved during the ET.

Nine patients (43%) referred from PH-centers other than Heidelberg did not attend the visit after 15 weeks, mainly due to the long traveling distance. Results remained significant after multiple imputation of missing values for the 6MWD at 15 weeks using the explanatory variables as described in Methods. The improvement in 6MWD ranged from 71 to 79 meters in the imputation replications. Patients who did not attend the assessment after 15 weeks had a significantly higher peak oxygen consumption at the AT (716 ± 139 vs. 543 ± 161 ml/min; *P *= 0.038) and a higher improvement in 6MWD (79 ± 62 vs. 50 ± 24 meters; *P *= 0.156) after 3 weeks. They did not differ significantly in other parameters such as demographics or parameters of right heart catheterization. After Bonferroni adjustment, improvement in 6MWD remained statistically significant.

**Figure 2:**
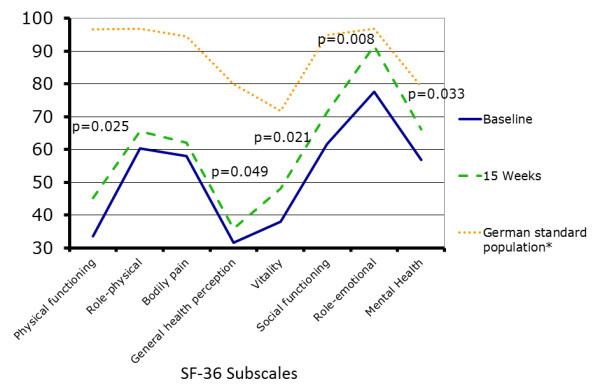
**Mean short-form Health Survey Questionnaire (SF-36) scores for quality of life subscales before and after exercise training**. At baseline (straight line), mean SF-36 scores were significantly reduced in comparison to respective values from a normal population (dotted line). After 15 weeks (dashed line), 5 scales of the SF-36 questionnaire improved significantly: physical functioning, general health perception, social functioning, mental health and vitality. *P*-values are indicated vs. baseline. No significant improvement was found for role emotional (ROLEM), role physical (ROLPH) and bodily pain (PAIN) after training. With Bonferroni adjustment, values of *P *< 0.005 were considered statistically significant. Data were available and were included in the analyses for 21 patients studied at baseline and for 18 patients studied at 15 weeks.

ET also significantly improved QoL parameters indicated by the SF-36 subscale scores for physical functioning (*P *= 0.025), general health perception (*P *= 0.049), social functioning (*P *= 0.008), mental health (*P *= 0.033) and vitality (*P *= 0.021) (Figure 2, Table 2).

**Table 2:** Efficacy parameters

Characteristic	Baseline (*n *= 21)			3 weeks (*n *= 21)			*P*-value	15 weeks (*n *= 14)			*P*-value
**6-minute walking distance, m**											
Mean change	386	±	121	425	±	118		447	±	139	
				64	±	17	< 0.001^a^	71	±	35	0.003
95% CI for the difference to baseline				42	-	85		48	-	95	

**Quality of life questionnaire SF36, score**											
Physical functioning	33.5	±	20				0.025^b^	45.2	±	20	0.025^b^
Role-physical	60.4	±	36				0.317	65.6	±	23	0.317
Bodily pain	58.1	±	33				0.260	62.2	±	31	0.260
General health perception	31.6	±	15				0.049^b^	35.9	±	14	0.049^b^
Vitality	38.1	±	14				0.021^b^	48.1	±	18	0.021^b^
Social functioning	61.8	±	30				0.008^a^	71.4	±	23	0.008^a^
Role-emotional	77.6	±	22				0.115	91.6	±	14	0.115
Mental Health	56.8	±	21				0.033^b^	66.2	±	16	0.033^b^

**Cardiopulmonary exercise testing**											
Peak VO_2_/kg, mL/min/kg	11.8	±	3.4	13.6	±	3.4	0.010^b^	14.1	±	3.5	0.008^a^
Peak VO_2_, ml/min	822	±	252	882	±	218	0.011^b^	887	±	139	0.007^a^
EqCO_2 _at AT, ml/min	41.3	±	8.9	41.3	±	9.5	0.529	42.8	±	7.1	0.138
VO_2 _at AT; ml/min	619	±	172	689	±	207	0.013^b^	681	±	207	0.012^b^
Oxygen pulse, (mL/min)/min^-1^	6.7	±	1.6	6.8	±	1.8	0.127	6.3	±	0.4	0.260
HR rest, min-1	85	±	14	77	±	12	0.0001^a^	78	±	16	0.045^b^
HR max, min-1	122	±	21	127	±	18	0.076	140	±	20	0.005^a^
RRsys rest, mmHg	117	±	17	114	±	15	0.092	111	±	15	0.653
RRdiast rest, mmHg	75	±	8	71	±	9	0.038^b^	72	±	8	0.464
RRsys max, mmHg	149	±	28	154	±	18	0.743	153	±	18	0.002^a^
RRdias max, mmHg	85	±	13	88	±	14	0.292	88	±	12	0.083
Oxygen saturation rest, %	94.7	±	3.0	96.7	±	3.2	0.266	96.4	±	2.8	0.421
Oxygen saturation max, %	89.8	±	5.6	90.8	±	5.8	0.052	91.7	±	6.3	0.018^b^
sPAP rest, mmHg	52	±	14	47	±	9	0.005^a^	50	±	10	0.210
sPAP max, mmHg	85	±	26	87	±	20	0.959	89	±	14	0.320
Workload max, W	55	±	19	67	±	23	0.007^a^	65	±	20	0.008^a^
Borg Scale, score	16	±	2	15	±	2	0.171	15	±	1	0.105

**Laboratory parameters**											
C-reactive protein, mg/l	15.2	±	22.5	8.3	±	9.7	0.069	6.6	±	5.3	0.071
Leucocytes, 10^9^/l	9.2	±	7.5	10.0	±	12.9	0.071	8.6	±	7.6	0.177
NT-proBNP, pg/ml	1928	±	4623	1101	±	1380	0.164	2321	±	2671	0.646

Values are mean ± standard deviation. ^a^*P *< 0.01, ^b^*P *< 0.05 in comparison to baseline; *P*-values are the same for absolute values and differences. 6-minute walking distance data were analyzed by the two-sided Student *t*-test; cardiopulmonary exercise testing data were analyzed by the Wilcoxon test. CI: confidence interval; SF-36: short-form Health Survey Questionnaire; VO_2_/kg: maximum oxygen consumption/kg; EqCO_2_: ventilatory equivalent for carbon dioxide; HR: heart rate; RRsys: systolic blood pressure; RRdiast: diastolic blood pressure; sPAP: systolic pulmonary arterial pressure; NT-proBNP: N-terminal prohormone of brain natriuretic peptide.

**Figure 3:**
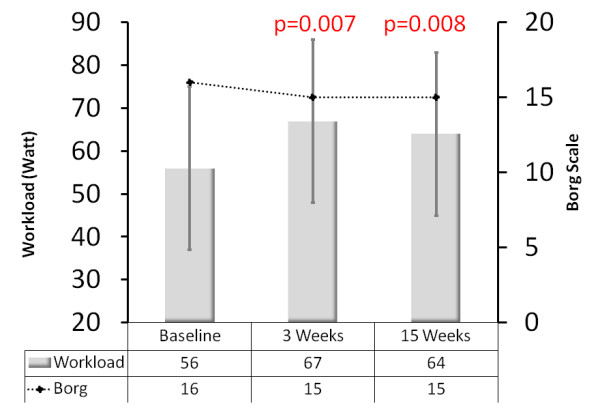
**Workload compared to the Borg scale**. The figure shows a significant increase in workload after 3 and 15 weeks (*P *= 0.007 and *P *= 0.008). The Borg scale remained unchanged (*P *= 0.171 and *P *= 0.105, respectively) although significantly higher workloads and higher heart rates during exercise were attained. Values are indicated as the mean, and the bars represent standard deviation. The columns represent workload (measured in Watt) and the dotted line the Borg Scale.

Mean peak oxygen consumption, peak oxygen consumption/kg body weight, oxygen consumption at the anaerobic threshold, and oxygen saturation at maximal workload during cardiopulmonary exercise testing increased significantly from baseline to 3 weeks and to 15 weeks (Table 2). Heart rate at rest significantly decreased after 3 and 15 weeks. After 15 weeks patients achieved significantly higher workloads with an increase of maximal heart rate during cardiopulmonary exercise testing and maximal systolic blood pressure. The Borg scale remained unchanged although significantly higher workloads and higher heart rates during exercise were attained (Table 2 Figure 3). After 3 weeks of ET, mean diastolic blood pressure and sPAP at rest were significantly reduced (Table 2). C-reactive protein reduced in trend, but not significantly, after 3 and 15 weeks, whereas N-terminal prohormone of brain natriuretic peptide (NT-proBNP) plasma-levels remained unchanged. Seven patients improved their WHO-FC after 15 weeks; five of them were reclassified as WHO-FC IV. Nevertheless, the change in WHO-FC after 3 weeks and after 15 weeks compared to baseline was not significant (*P *= 0.058 and *P *= 0.096, respectively), possibly due to the small sample size.

### Adverse events

During the 3-week in-hospital training, three patients had adverse events as follows: gastrointestinal infection with diarrhea (*n *= 1) and respiratory infections (*n *= 2). In all three patients the infection was treated successfully so that they were able to continue the training program after one or two weeks. All other patients (81%) tolerated the ET well. There were no signs of clinical worsening or right heart failure during the in-hospital program.

### Follow-up and survival

**Figure 4:**
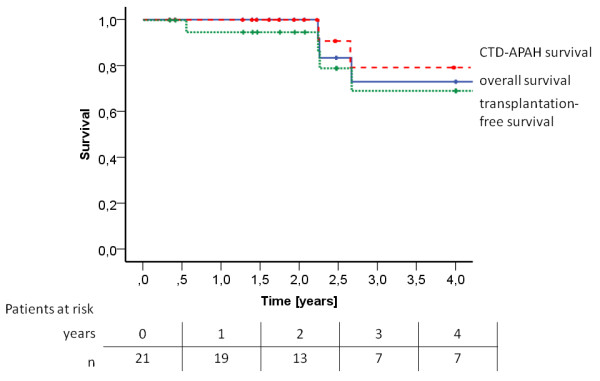
**Survival by Kaplan-Meier analysis**. Within a follow-up period of 2.9 ± 1.9 years (25 ± 13 months), two patients died due to pulmonary hypertension (PH), one died due to carcinoma, and one patient underwent lung transplantation. The dashed line indicates mortality due to connective tissue disease-associated pulmonary arterial hypertension (CTD-APAH), the straight line indicates overall survival, and the dotted line indicates transplantation-free survival. The Kaplan-Meier PAH-survival rate was 100% after 1, and 2 years, and 80% after 3 years. The overall survival rate was 100% after 1, and 2 years, and 73% after 3 years. The transplantation-free survival rate was 95% after 1 and 2 years.

Follow-up data were obtained after 2.9 ± 1.9 years (Figure 4). One patient had been listed for lung transplantation before starting the rehabilitation program. She improved her walking distance by about 88 meters after 3 weeks, but did not perform the examination after 15 weeks due to the long traveling distance. However, she required lung transplantation 6 months later. Between 2.8 and 5.0 years after in-hospital rehabilitation, two patients died due to PAH and right heart failure, and one patient died due to cancer of unknown primary origin. The Kaplan-Meier PAH-survival rate was 100% after 1 and 2 years, and 80% after 3 years (Figure 4). The overall survival rate was 100% after 1 and 2 years, and 73% after 3 years. The transplantation-free survival rate was 95% after 1 and 2 years (Figure 4).

ET was continued over 3 years by 11 patients (61%). They reported a mean ET-duration of 29 ± 18 minutes at 4 ± 2 days/week. Among the 11 patients, 44% continued bicycle ergometer training, 17% dumbbell-training, 33% walking and 22% alternative training, such as gymnastics. Four patients (22%) combined two training items and three patients (17%) combined three items. Six patients (33%) stopped exercise training for the following reasons: pain, presyncopes, no permanent instruction and confidence with general state of health.

## Discussion

This is the first prospective clinical trial investigating short- and long-term effects of ET as an add-on to PAH-targeted medication in patients with severe CTD-APAH. The results of the study suggest that ET can significantly improve prognostic relevant parameters such as exercise capacity and QoL in this condition, and has an excellent long-term survival of 100% after 2 years and 80% after 3 years.

The results represent an important source of data on survival, exercise capacity and QoL in patients with CTD-APAH treated with ET. The 6MWD, QoL questionnaire (SF-36), peak oxygen consumption, oxygen consumption at the AT, maximal oxygen saturation and maximal workload increased significantly with ET after 3 and 15 weeks. RRdiast and sPAP significantly decreased after 3 weeks. This may be due to an improvement in endothelial dysfunction. Nevertheless, due to the limited number of patients we cannot exclude that these improvements occurred coincidentally. The Borg scale remained unchanged although significantly higher workloads and higher heart rates during exercise were attained. These results are in line with previous studies of ET in patients with other forms of PH/PAH [13-15]. There are few data on exercise training in CTD. In patients with SSc but not PAH, ET has improved QoL parameters (SF-36) and heart rate at rest [19], 6 MWD [18] and muscle strength [17]. Quality of life and peak VO_2 _have also been significantly ameliorated by training in patients with SLE without PAH [16].

### Survival of patients with CTD-APAH after training

The Kaplan-Meier overall survival rate of 100% after 1 and 2 years and 73% (80% PH-related survival) after 3 years, using ET as add-on to optimized PAH-targeted medical treatment, can be considered a positive outcome in CTD-APAH. Despite optimized PAH-targeted therapy, the Reveal registry showed a 1-year survival in patients with SSC-APAH of only 82% vs. 84% in SLE-APAH, 88% in MCTD-APAH and 93% in IPAH [7]. In two trials of bosentan in CTD-APAH-patients, the Kaplan-Meier estimates of survival were 85.9% and 73.4% after 1 and 2 years, respectively [23]. The 3-year survival rate in SSc-APAH was 60% vs. 78% in IPAH [8]. Similar patterns have been seen in the UK registry [9] and the French registry [24,25] and in several further studies [26]. These data indicate that patients with CTD-APAH represent a high-risk subgroup of patients with PAH. The CTD-APAH-cohort assessed in this study had been severely affected with a mean 6MWD of 386 ± 121 meters at baseline despite double or even triple PAH-targeted therapy in 62% of patients, and is therefore comparable to the cohorts described previously. We cannot exclude that we selected highly motivated and compliant patients with fewer outcome limitations. In addition, the number of patients in our study was quite small. Nevertheless, ET may have improved survival by ameliorating prognostic relevant factors such as QoL, exercise capacity and oxygen consumption. Since heart rate at rest, RRdiast and peak oxygen consumption significantly improved, ET possibly improved right ventricular reserve [14]. Similar effects of ET have been seen in patients with IPAH and CTEPH and right heart failure [14,15].

### Improvement in 6-minute-walking distance

The 6MWD has been used as primary endpoint in many randomized controlled clinical trials in PAH [1], and correlates with prognostic relevant clinical parameters such as WHO-FC in patients with SSc-PAH [6]. However, the test is not sufficiently validated in CTD-APAH and in patients with rheumatic diseases in particular, it is influenced by comorbidity and musculoskeletal limitations [6,27]. This has been taken as explanation for the fact that 6MWD in patients with CTD-APAH is at best only minimally improved by PAH-targeted medication. In randomized controlled trials, sildenafil is the only drug to have been associated with a significant increase in 6MWD in patients with CTD-APAH (in many other trials no subgroup analysis for this disease has been performed). Therefore, a mean increase of almost 70 meters after 3 and 15 weeks ET is unexpectedly high. The absence of a non-trained placebo group may be considered a limitation of the study, with concern that some of the improvements were due to the so-called placebo effect, rather than efficacy of ET. However, previously reported placebo-controlled PAH studies of bosentan or other PAH-specific treatments have not shown any clinically relevant improvements in the placebo groups. In fact, they have consistently shown a continuous decline in 6MWD. Therefore, the results of this study suggest that, especially in CTD-APAH-patients with known high coincidence of musculoskeletal limitations, ET as an add-on to PAH-targeted medication may be useful, and possibly even more effective in improving exercise capacity than medical treatment. Further randomized trials are needed to clarify this question.

### Limitations

The results of this prospective study are promising and our data provide a good rationale for future studies of exercise training in patients with CTD-APAH. However, the positive results may also be influenced by the small sample size. Larger, randomized controlled and multicentric studies are needed to determine the effectiveness of exercise training in CTD-APAH. The effects of ET after 15 weeks may be biased due to the missing values of about 43% of patients who did not perform the last follow-up visit. A comparison between patients who completed the 15 week assessment and those who did not revealed that patients who dropped out had even better improvement in efficacy parameters after 3 weeks in-hospital rehabilitation. This suggests that they possibly did not attend because they felt better. Feedback from patients during follow-up visits supports this assumption.

It is a general issue of rehabilitation programs that the therapy cannot be performed in a blinded fashion and that a referral bias towards highly motivated patients with a better outcome may occur. Further studies are necessary to determine the effects of training programs on outcome in patients with PH.

## Conclusion

This is the first trial investigating exercise training in CTD-APAH as an add-on to optimized medical therapy. The results indicate that ET is effective in CTD-APAH and may improve work capacity, QoL and further prognostic relevant parameters, and possibly improves the 1-, 2- and 3-year survival rate. Further randomized controlled studies are needed to confirm these results.

## Abbreviations

APAH: associated pulmonary arterial hypertension; AT: anaerobic threshold; CI: confidence interval; CTD: connective tissue disease; EqCO_2_: ventilatory equivalent for carbon dioxide; ET: exercise training; FEV1%VC: forced expiratory volume in one second/vital capacity; IPAH: idiopathic pulmonary arterial hypertension; MCTD: mixed connective tissue disease; 6MWD: six minute walking distance; NT-proBNP: N-terminal prohormone of brain natriuretic peptide; PAH: pulmonary arterial hypertension; PCWP: pulmonary capillary wedge pressure; PH: pulmonary hypertension; pO2: partial oxygen pressure; QoL: quality of life; REVEAL-Registry: Registry to Evaluate Early and long Term PAH Disease Management; RRdiast: diastolic systemic blood pressure; RRsys: systolic systemic blood pressure; SD: standard deviation; SF-36: short form Health Survey Questionnaire; SLE: systemic lupus erythematosus; sPAP: systolic pulmonary arterial pressure; SSc: systemic sclerosis; TLCO: carbon monoxide transfer factor; VCO2: carbon dioxide output; VE: ventilation; VO2/heart rate: oxygen pulse; VO2: oxygen uptake; WHO-FC: World Health Organization functional class.

## Competing interests

The authors declare that they have no competing interests.

## Authors' contributions

EG, CN and NE were responsible for designing the study, and conducting and analyzing the results and were directly involved in data collection. FM, FP and ML were directly involved in acquisition of data. CF was directly involved in analyzing and interpreting the results. NB, CF, FS, GS, FR, HT, MH, HJS and SW were principle investigators directly involved in data collection. All authors were involved in the writing of the manuscript and saw and approved the final version of the paper.
